# Microgeographical Variation in *Dirofilaria immitis* Prevalence in Dogs in Suburban and Urban Areas of Rio De Janeiro, Brazil

**DOI:** 10.3390/vetsci12010003

**Published:** 2024-12-27

**Authors:** Marianna Laura Elis Chocobar, Elizabeth Moreira dos Santos Schmidt, Ângelo Joel Ferreira Mendes, Paul Christopher Duncan Johnson, William Weir, Rossella Panarese

**Affiliations:** 1 School of Veterinary Medicine and Animal Science (FMVZ), São Paulo State University (UNESP), Rua Prof. Dr. Walter Maurício Corrêa, s/n, Botucatu 18618-681, SP, Brazil; laura.elis@unesp.br; 2 School of Biodiversity, One Health and Veterinary Medicine, College of Medical, Veterinary and Life Sciences, University of Glasgow, Garscube Estate, Glasgow G61 1QH, UK; a.mendes.1@research.gla.ac.uk (Â.J.F.M.); paul.johnson@glasgow.ac.uk (P.C.D.J.); willie.weir@glasgow.ac.uk (W.W.); rossella.panarese@glasgow.ac.uk (R.P.)

**Keywords:** canine heartworm, mosquito-borne infection, one health, filarial diagnostics, suburban areas, urban areas, economic disparity, environmental degradation

## Abstract

Dirofilariosis, a mosquito-borne disease caused by *Dirofilaria immitis* and *Dirofilaria repens*, affects canids, felids and occasionally humans. Recent evidence suggests that *D. immitis* prevalence is rising in the canine populations in several areas of Brazil, even those historically considered to be non-endemic, highlighting the need for ongoing surveillance. However, prevalence studies are frequently based on inference from single diagnostic methods, and it is acknowledged that this may lead to biases and an underestimation of the disease situation. Thus, this cross-sectional study aimed to assess the prevalence of *Dirofilaria* spp. infection in dogs and cats across suburban and urban areas in Rio de Janeiro, Brazil. The investigation was conducted using a multi-diagnostic approach with binomial generalised linear models (GLMs) applied to investigate the association between the occurrence of infection and potential disease determinants. Blood samples from 497 dogs and 107 cats, collected at a veterinary mobile neutering unit, underwent parasitological, serological and molecular testing to detect *Dirofilaria* spp. infections. Among the dog samples, an overall 7.44% tested positive for *D. immitis* infection, while *D. repens* was not detected. In contrast, no cat samples tested positive for either parasite species. Prevalence was notably higher in suburban areas (14.47%) than in the urban area of Rio de Janeiro (6.17%) (*p* = 0.003). Dogs aged over two years had a higher risk of being infected (*p* < 0.001), while features such as breed, sex, size, coat type, use of macrocyclic lactones or access to outdoor spaces were not found to be predictive factors. This study reinforces the evidence that the prevalence of *D. immitis* is increasing in areas previously considered to have low prevalence or to be non-endemic. It also suggests that environmental and socioeconomic factors may play a key role in determining the current epidemiological status of this parasite. These findings highlight the need for increased monitoring in high-risk areas, targeted prevention and public awareness to address this emerging One Health concern.

## Simple Summary

This study investigated the prevalence of *Dirofilaria immitis*, a zoonotic mosquito-borne parasite, in dogs and cats from suburban and urban areas of Rio de Janeiro, Brazil. The investigation was conducted using a multi-test approach with sophisticated statistical analyses applied to determine potential risk factors associated with infection. The main results of the study demonstrate an increased prevalence of *D. immitis* infection in the canine population in Rio de Janeiro in relation to previous studies, which was significantly higher in suburban compared to urban areas. This study not only indicates that the parasite is now established in regions historically considered non-endemic for the infection, but also that dogs living in those areas, potentially characterised by environmental degradation and social deprivation, are at elevated risk of contracting the infection.

## 1. Introduction

Dirofilariosis, a zoonotic mosquito-borne disease, is caused by filariids of the Onchocercidae family and affects several animal species, including humans. Two species within the *Dirofilaria* genus are of primary medical and veterinary importance: *Dirofilaria immitis* (Leidy, 1856), the causative agent of canine Heartworm Disease (HWD) and Heartworm-Associated Respiratory Disease (HARD) in cats, and *Dirofilaria repens* Raillet and Henry, 1911, responsible for Subcutaneous Dirofilariosis (SCD) [1,2]. Cats are considered imperfect hosts due to the low and transient microfilaraemia, thus hindering onward transmission of *D. immitis* [3]. Dogs and other canids form a reservoir of infection and play a key role in the epidemiology of the disease, especially when competent mosquito-vector species are also present in the same geographical area [4]. Several mosquito species belonging to the genera *Ochlerotatus*, *Culex*, *Anopheles*, and *Aedes* are considered putative or competent vectors of *Dirofilaria* spp., and their relative importance varies in different parts of the world [5]. In Brazil, a number of species have been recognised as competent vectors of *D. immitis*, with *Aedes scapularis*, *Aedes taeniorhynchus*, and *Culex quinquefasciatus* considered the most important in the state of Rio de Janeiro, southeast Brazil [6,7].

In the past ten years, five prevalence studies on dogs and two involving cats have been undertaken in the city of Rio de Janeiro, one of Brazil’s five largest metropolitan centres. Over the same period, seven studies on dogs alone have been conducted in Baixada Fluminense, a suburban region which is continuously growing as a result of the urban expansion of the greater Rio de Janeiro metropolitan area [8,9]. The latest study in Rio de Janeiro has reported prevalences of 7.0% and 0.9% in dogs and cats, respectively [10]. Studies in the suburban region, historically regarded as non-endemic for dirofilariosis [11], more recently reported an overall prevalence of 2.85% [11] and later 13.20% [12] in canine populations. There is currently no published data on the prevalence of infection with *Dirofilaria* spp. in cats in this region. Furthermore, the studies conducted in both the suburban region and Rio de Janeiro city relied exclusively on a single diagnostic method, a critical limitation that might have led to a potential underestimation of the true prevalence [2,9].

Knowledge gaps persist regarding *Dirofilaria* spp. distribution in all Brazilian regions, likely compounded by substantial under-reporting of the disease due to limited diagnostic methods employed [9]. There is also low adherence to preventive measures, i.e., the year-round administration of macrocyclic lactones [9,12,13]. Most of the research in Brazil to date has focused on *D. immitis* [10,13], which, to this day, is considered the only endemic *Dirofilaria* species in the country, despite there having been two documented cases of *D. repens*, separated by an interval of one hundred years [14,15]. In addition, there is evidence of a new *Dirofilaria* species circulating in the Americas and other regions [16,17,18,19,20,21,22], and therefore comprehensive studies using discriminatory parasite genetic markers are now required.

The urban and suburban areas of Rio de Janeiro are associated with dramatically different environmental and socioeconomic factors, and it is important to understand whether there is an appreciable difference in the epidemiology of *D. immitis* in these areas. To overcome some limitations of earlier studies, the aim of the present work was to undertake a combined parasitological, serological, and molecular approach to: (i) update the prevalence data on *Dirofilaria* spp. in canine and feline populations in Rio de Janeiro metropolitan areas and (ii) investigate the association between the occurrence of infection and potential disease determinants using binomial generalised linear models (GLMs).

## 2. Materials and Methods

### 2.1. Sample Collection

All procedures carried out in this experiment were in accordance with international ethical standards and current legislation on animal protection and were approved by the Ethics Committee of the School of Veterinary Medicine and Animal Sciences (CEUA0544/2023), São Paulo State University (FMVZ-UNESP), Brazil.

**Figure 1:**
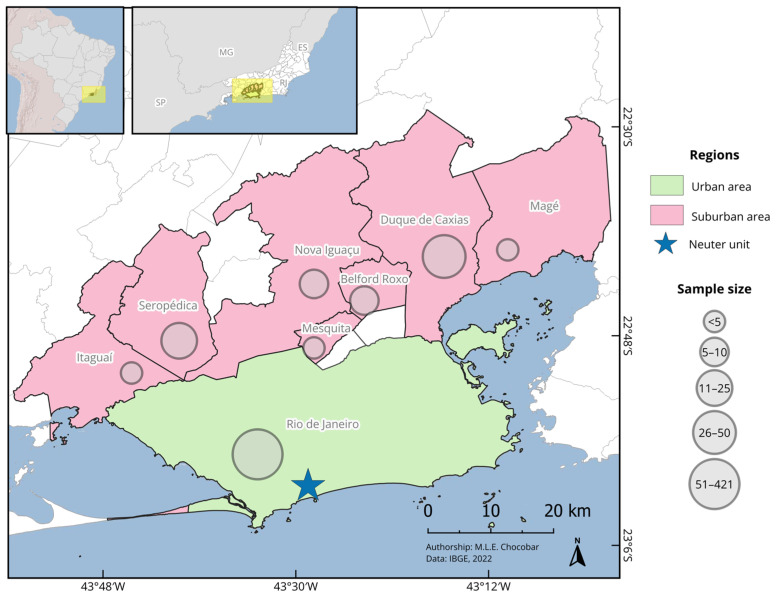
Study areas in suburban (pink) and urban (green) regions of Rio de Janeiro, Brazil. The sample size for dogs is indicated by the size of the circles. This figure was created using QGIS Geographic Information System (Version 3.36).

Samples were collected between 19 February and 7 March 2024, at a veterinary mobile spay and neuter unit located in Recreio dos Bandeirantes (23°01′19″ S, 43°29′18″ W), a neighbourhood in the west of Rio de Janeiro city, Brazil (Figure 1). Animals underwent clinical examination and blood and serum sample collection prior to neutering. A qualified veterinarian ensured that dogs and cats enrolled in the study showed good health status (i.e., absence of clinical signs at the clinical examination), were over six months old, and came from any area within the state of Rio de Janeiro. Additionally, owners had to provide proof of low-income status to be eligible for the neutering programme. Rio de Janeiro city was classified as the urban area, while all municipalities within Baixada Fluminense were classified as the suburban area (Figure 1).

The sample size was calculated using Epi Info 2000 (CDC, Atlanta, GA, USA) based on the canine and feline population estimates for urban and suburban areas, with a 95% confidence interval, a desired absolute precision of ±5%, and the expected prevalence of heartworm infection: 7% in urban dogs (n = 100), 2.85% in suburban dogs (n = 43), and 0.9% in urban and suburban cats (n = 14) [10,11].

**Figure 2:**
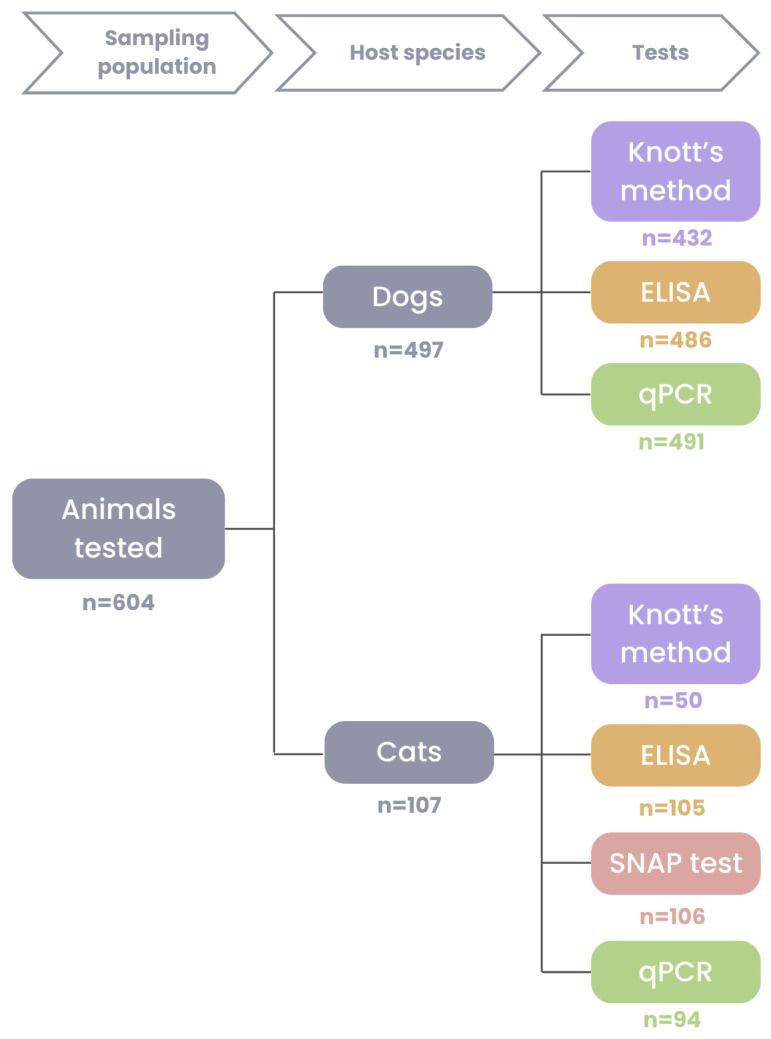
Flowchart indicating the number of samples processed by the modified Knott’s method (purple), ELISA (orange), SNAP test (pink) and duplex qPCR (green) for each animal species.

Blood samples were obtained from 497 dogs (76 from the suburban area and 421 from the urban area) and 107 cats (21 from the suburban area and 86 from the urban area), by cephalic or jugular venipuncture. Sample aliquots of 0.5 mL each were transferred into tubes containing EDTA (Ethylenediaminetetraacetic Acid) and stored at −80 °C for molecular analysis, while 1 mL aliquots of the remaining volume were refrigerated at 4 °C for parasitological examinations. Serum samples of 2.5 mL were collected in plain tubes, centrifuged at 1252 g for ten minutes, and the sera were harvested, stored in Eppendorf microtubes (Eppendorf, Hamburg, Germany) and kept at −80 °C. Due to sample collection limitations, it was not always possible to collect sufficient blood for all tests from each animal. When prioritising, preference was given firstly to plain tubes for serological tests, secondly to EDTA aliquots for molecular analysis, and thirdly to EDTA aliquots for parasitological tests, according to each test’s sensitivity [2]. The exact number of canine and feline samples tested for each method is shown in Figure 2.

### 2.2. Parasitological and Serological Screening

Knott’s modified method [23,24] was performed with EDTA blood and all microfilariae were morphometrically characterised using a 20× objective lens on an Olympus BX60 microscope (Olympus Corporation, Tokyo, Japan) and identified using the previously published morphological keys [25].

Serum samples were analysed using the MegaELISA^®^ DIRO Antigen test (Diagnostik Megacor, Hörbranz, Austria, Lot ME-D-026), following the manufacturer’s instructions. This test detects current infections by identifying circulating *D. immitis* antigens. Samples were analysed without prior heat or acid treatment to reduce the risk of false positive results due to the potential presence of *Acanthocheilonema reconditum* which was previously reported in the same geographical area [11,26]. Plates were read using a Multiskan™ FC Microplate Photometer (Thermo Scientific™, Waltham, MA, USA) at 450 nm. Absorbance values ≥ 0.1 were considered positive, while values < 0.1 were considered negative for *D. immitis* antigen.

Feline serum samples were also tested with the FASTest^®^ HW Antigen (Diagnostik Megacor, Hörbranz, Austria, Lot 7126B) test, a rapid SNAP test targeting *D. immitis* antigens, following the manufacturer’s instructions without prior heat or acid treatment, due to the low volume of serum sample available.

### 2.3. Molecular Analysis

Total DNA was extracted from samples placed in tubes with EDTA using QIAwave DNA Blood & Tissue kit^®^ (Qiagen^®^, Hilden, Germany, Lot 178018171), following the manufacturer’s instructions. Extracted DNA was stored at −80 °C.

First, a duplex real-time PCR (qPCR) was performed to simultaneously detect *D. immitis* and *D. repens* DNA, following the previously described methodology [27]. The positive control material for *D. repens* was an adult parasite found in a nodular mass on a dog’s nose from the United Kingdom [28]. The *D. immitis* positive control was an adult worm found in a female dog’s abdomen during neutering surgery in Rio de Janeiro, Brazil, collected in the course of the current study, and morphologically and molecularly identified as an immature male.

All reactions were performed in a QuantStudio5^®^ thermocycler (Applied Biosystems^®^, Waltham, MA, USA). Samples with melting curves of 75.7 ± 0.3 °C (mean, SD) were considered to be positive for *D. immitis* and those with melting curves of 70.0 ± 0.7 °C were deemed positive for *D. repens* [27].

Samples providing inconclusive qPCR results due to sample inhibition were re-tested using conventional PCR (cPCR) targeting either the 12S rDNA (n = 3) or cox1 (n = 1) loci as described elsewhere [28]. The cox1 target was used for one sample after it tested negative for 12S. Reactions were performed in a final volume of 17 µL, consisting of 0.1 µL of HotStar Taq^®^ DNA polymerase (Qiagen^®^, Hilden, Germany), 2 µL of 10× reaction mix, 0.16 µL of dNTP, 10.74 µL of nuclease-free water and 2 µL of each primer [28]. Amplicons were then purified using QIAquick^®^ PCR Purification Kit (Qiagen^®^, Hilden, Germany), sequenced (Sanger sequencing) and compared with the GenBank non-redundant nucleotide database using the Basic Local Alignment Search Tool (BLAST).

To identify potential co-infections with the filarial nematode *Acanthocheilonema reconditum*, samples that tested positive for *D. immitis* by at least one diagnostic method (i.e., modified Knott test, ELISA, duplex qPCR or cPCR) were further analysed by cPCR using species-specific primers for *A. reconditum* targeting the 5.8S-ITS2-28S genes [29]. Reactions were performed in a final volume of 17 µL, consisting of 0.1 µL of HotStar Taq^®^ DNA polymerase (Qiagen^®^, Hilden, Germany), 2 µL of 10× reaction mix, 0.16 µL of dNTP, 10.74 µL of nuclease-free water and 2 µL of each primer.

### 2.4. Risk Factors Data Collection

To evaluate the risk factors associated with *D. immitis* infection, prior to sample collection, all owners signed a consent form and answered an ad hoc questionnaire regarding the following information: (i) animal identification data (i.e., species, breed, sex, age, size, coat colour, coat length, and whether it had access to outdoor spaces), and (ii) data of epidemiological relevance (i.e., place of residence, use macrocyclic lactones in 2023, and presence of clinical signs associated with HWD).

To prevent overfitting in the statistical model and ensure a more robust analysis, certain categories were merged. Breed was classified into two categories: mixed or specific. Age was grouped into three categories: younger than 2 years old, 2–4 years old, and older than 4 years. Coat colour was simplified into dark (comprising black and grey dogs) and light (including white and caramel dogs), while coat length was divided into categories of short or medium-long. Access to outdoor spaces was categorised as indoor, encompassing dogs that live indoors and those regularly walked with a leash, or outdoor. Time spent in the current residence was classified as short, less than one year, or long, more than one year. Dog size was divided into small (<10 kg), medium (10 to 20 kg), and large (>20 kg) classes.

Although all samples were collected at the same spay and neuter unit, dogs and cats came from eight different municipalities of Rio de Janeiro State. These represented the urban area of Rio de Janeiro city and the suburban area, constituted of Belford Roxo, Duque de Caxias, Itaguaí, Magé, Mesquita, Nova Iguaçu, and Seropédica [30].

### 2.5. Statistical Analysis

Descriptive statistics were computed, and plots were generated to illustrate general data patterns. Mean, standard deviation, median, and interquartile range were calculated for continuous variables. Frequencies and percentages were calculated for categorical variables.

Binomial generalised linear models (GLMs) were used to analyse the association between *D. immitis* infection status and the following categorical variables: region (suburban area vs. urban area), age (<2 years old, 2–4 years old, >4 years old), breed (mixed vs. specific), sex (female vs. male), size (small, medium, large), coat colour (dark vs. light), coat length (short vs. medium-long), access to outdoor spaces (indoor vs. outdoor), time in place (short vs. long), and use of macrocyclic lactones (ML) in 2023 (no vs. yes). The GLMs were built in two stages allowing univariable and multivariable analyses.

GLM coefficients were exponentiated to obtain the odds ratio (OR). The ‘confint’ R function was used to obtain 95% confidence intervals. Likelihood ratio tests implemented in the drop1 R function were used to obtain the *p*-value. Variables with a *p*-value < 0.2 were selected for multivariable analyses.

At multivariable analyses, a stepwise backward selection procedure was followed by removing variables with *p* > 0.05 until all variables were significant at *p* < 0.05. Adjusted OR, confidence interval, and *p*-value were reported for each variable in the final multivariable model. All analyses were performed using the R statistical software (version 4.3).

## 3. Results

**Figure 3:**
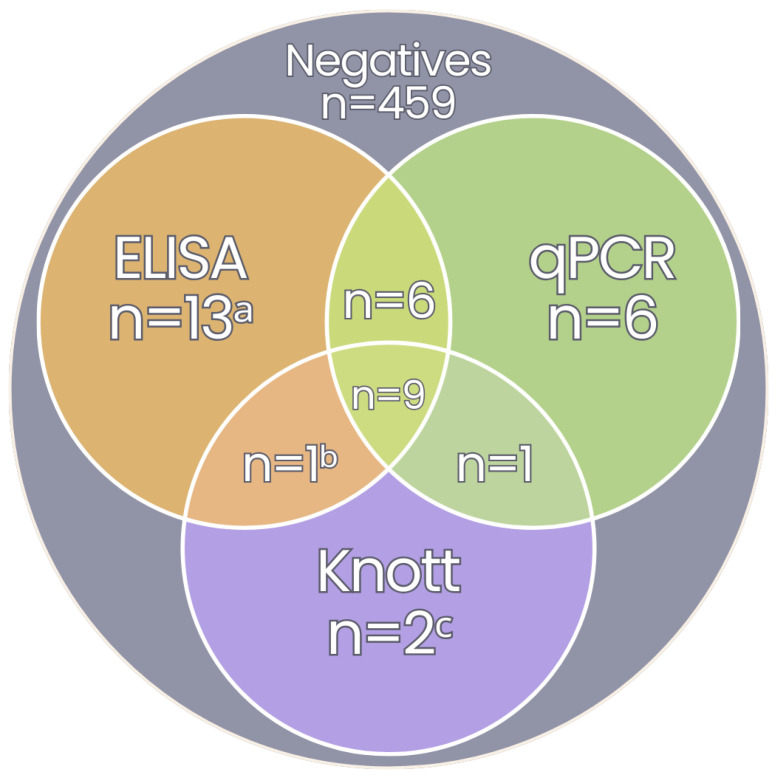
Venn diagram showing the distribution of 37 dog samples positive for *D. immitis* and one sample positive for *Acanthocheilonema reconditum* across different diagnostic methods: negative for all tests (grey), ELISA (orange, n = 29), Duplex qPCR (green, n = 22) and Knott’s modified test (purple, n = 13), including their overlaps. ^a^ Three samples were analysed exclusively by this method; ^b^ Sequenced as *Dirofilaria immitis* (100% identity); ^c^ One sample was sequenced as *Dirofilaria immitis* (99.63% identity) and the other as *Acanthocheilonema reconditum* (100% identity).

Among the 497 dog samples analysed, 7.44% (37/497) tested positive for *D. immitis* infection in at least one of the three diagnostic tests performed (Figure 3), yet none of these dogs showed clinical signs of dirofilariosis. In contrast, none of the 107 cat samples tested positive for *Dirofilaria* spp. using any of the four diagnostic methods applied.

Microfilariae (mfs) were found in 13 of 432 dog samples (3.01%) at a median concentration of 140 mf/mL, with an average length of 288 ± 5.07 µm and width of 5.90 ± 0.14 µm. One of these samples was later identified as positive to A. reconditum mfs through Sanger sequencing. The detection of *D. immitis*, determined by the modified Knott’s method, was lower than initially expected (2.77%). The ELISA test revealed the highest detection rate among the diagnostic tests, with 29 of 486 dog samples (5.97%) testing positive (Supplementary Table S1).

In total, 22/491 samples (4.48%) were found to be positive for *D. immitis* using Duplex qPCR, with mean Ct values of 21.00 ± 1.20 and melting curve temperatures of 76.20 ± 0.09 °C. Additionally, among the four inconclusive qPCR results, one was confirmed to be negative, while the other three were determined to be positive for *D. immitis*. Thus, the overall detection of *D. immitis* by molecular methods was 4.89% (24/491) (Supplementary Table S1). None of the samples tested positive for *D. repens* by molecular methods, and no co-infections with A. reconditum were identified.

A higher prevalence of *D. immitis* (14.47%) was observed for the suburban area of Rio de Janeiro, compared to the urban area (6.17%), a difference which was statistically significant (*p* = 0.003, adjusted OR = 0.28 (95% confidence interval: 0.13–0.64)). Detailed disaggregated data is presented in Table 1.

The results of descriptive analysis together with univariable and multivariable logistic regression analyses regarding the potential risk factors for *Dirofilaria immitis* infection in dogs are shown in Table 2. Cats are not included in this statistical analysis as there were no positive cases of *Dirofilaria* spp.

Three evaluated risk factors were found to be predictors for *D. immitis* infection. Both univariable (*p* = 0.020) and multivariable (*p* = 0.003) analyses indicated that dogs residing in the urban area had a lower likelihood of infection than dogs from the suburban area.

Of all the assessed variables, age emerged as the most important risk factor (*p* < 0.001). Dogs aged between two and four years had approximately three times higher odds of being infected compared to those younger than two years. The odds of being infected was even greater in older dogs, with those over four years old showing a markedly higher risk, as reflected by the greater odds ratio in this age group (*p* < 0.001).

The analysis did not reveal any significant association between breed, sex, size, coat characteristics or access to outdoor spaces and the risk of infection (*p* > 0.05). Likewise, no significant association was observed between the use of macrocyclic lactones in 2023 and the presence of *D. immitis* infection (*p* = 0.183). The owners of 30 dogs and 17 cats did not provide information regarding preventive medication. Among the 467 dogs surveyed, 295 (63.17%) had not received any dose of macrocyclic lactones in 2023. The rate was higher for the 90 cats, as 72 (80%) received no prophylactic treatment in 2023. Adherence to a prophylactic regimen was considerably higher in the urban area (40.92%) than in the suburban area (15.79%).

## 4. Discussion

The 6.17% prevalence of *D. immitis* infection in dogs in the urban area of Rio de Janeiro determined in the present study was very similar to the figure of 7% reported by a recent serological study [10]. In contrast, the suburban areas had a significantly higher prevalence of 14.47%. This is a notable increase from the 2.85% reported in 2022 [11], which was based on parasitological and molecular methods, and the 2024 figure of 3.40% [31], obtained through parasitological and serological examinations. However, our findings align closely with the 13.2% suburban area estimated prevalence recently documented in 2024 using immunochromatography [12], suggesting an establishment of this parasite in this area over the last two years. These findings suggest a shifting dynamic in the distribution of *D. immitis* in Rio de Janeiro, indicating a potential trend toward the spread and establishment of the parasite in the suburban regions.

In contrast to many previous studies, the use of a multi-test strategy provides a more robust and comprehensive assessment. For instance, integrating the results of different diagnostic methods took the overall detection rate of *D. immitis* infection from as low as 2.77%, using only the modified Knott’s test, to 7.44% when serological, parasitological, and molecular results were combined. Although the study was not powered to assess diagnostic performance, it’s worth to mention that the modified Knott’s method showed the lowest sensitivity of all the tests (32.4%), followed by the molecular methods (59.5%) and the ELISA test (76.3%). This variation was anticipated and may be attributed to the different diagnostic targets and methodologies for each test, which together give rise to a difference in their sensitivities. This is often observed and may be compounded by biological factors such as stage of infection, presence of single sex infections, low or absent microfilaraemia, and presence of inhibitors of DNA amplification [2,4,13]. It is, therefore, prudent to undertake a multi-modal approach to *Dirofilaria* diagnostic testing.

Having accurate prevalence figures is highly important for both the scientific community and veterinary practitioners. Previously available data often relied on a single diagnostic method which may have led to under-reporting, a misrepresentation of prevalence, and missed opportunities for effective treatment and prevention campaigns. Nevertheless, each diagnostic tool can serve distinct purposes, particularly in areas where multiple filariid species coexist or where cryptic *Dirofilaria* species or subspecies may be circulating [20]. For example, molecular diagnostics should be more widely adopted, as its use remains limited in Brazil [9]. Over the past two decades, 50 cases of human dirofilariosis have been reported in Brazil, but only one benefitted from the use of molecular techniques, identifying a genetically variant form of *Dirofilaria* [17]. Similarly, genetic variants of *Dirofilaria* have been observed in dog populations in northern Brazil [16] and other regions of the Americas [20]. The broader application of molecular methods in both human and veterinary research is crucial for advancing our understanding of the epidemiology and genetic diversity of *Dirofilaria* spp., ultimately improving diagnosis, treatment, and prevention strategies.

The proportion of dogs with *D. immitis* infection (eight out of 32 dogs, 25%) in the municipality of Duque de Caxias, one of the suburban areas around the main capital, is much higher than was expected based on previously reported prevalences of 1.8% [11] and, more recently, 2.5% [31]. Duque de Caxias consists of four districts, with the namesake district housing most of the population due to its proximity to Rio de Janeiro [32]. Until its abrupt closure in 2012, the district was home to Latin America’s largest dumpsite, which has since been transformed into a ‘*favela*’. Grimly described by the Brazilian media as “*a neighbourhood thrown to waste*” [33], it now shelters nearly 40,000 former waste pickers who endure inadequate infrastructure, limited access to basic services, and pervasive poverty. Campos Elísios, the second-largest district, hosts Brazil’s second-largest oil refinery and faces significant environmental degradation and pollution [32,34]. The municipality has natural water sources, including Guanabara Bay and rivers like the Sarapuí, Iguaçu, Estrela, and Meriti, the latter bordering Rio de Janeiro city. Duque de Caxias experiences frequent flooding, especially in urban riverine areas [35,36], and only 89.3% and 59.3% of the population have access to sewage and water networks, respectively [30]. Limited flood prevention investments exacerbate social and environmental vulnerabilities in these areas, providing ideal conditions for mosquito proliferation [34,37]. The assessment of this structural issue can play a crucial role in preventing this and other vector-borne diseases.

To the best of our knowledge, this study provides the first prevalence estimate for *D. immitis* performed in Seropédica (suburban area), which highlights a research gap in this area. Until the late 60s, Seropédica’s land use was centred around extensive livestock and agricultural activities. However, its terrain, composed of sand, clay, and rocky soils, has made it an ideal location for over one hundred mining companies, which has led to severe environmental degradation, resulting in the formation of ‘*the sandy areas of Seropédica and Itaguaí*’. This area is characterised by sand deposits and pits filled by water of the Piranema Aquifer, which are located close to Seropédica’s urban areas and to the border with Rio de Janeiro city [8,38]. The rapid environmental changes and formation of artificial pools may well have facilitated mosquito proliferation in this area [39,40], potentially explaining the high prevalence of *D. immitis* infection in dogs. The stagnant water in these pools could be considered an ideal breeding ground for mosquitoes, thus increasing the risk of parasite transmission [37,41]. Having only obtained a single sample from the nearby municipality of Itaguaí for our study, and lacking any previous prevalence data, it was impossible to determine the prevalence of *Dirofilaria* spp. in this area. This underscores the urgent need for further work to clarify the extent to which the parasite can be found in this municipality. Stricter regulations on mining companies should be enforced to minimise the environmental impact of mining activities, with effective mitigation measures and compensation strategies put in place to protect local ecosystems and public health.

The absence of feline cases was not unexpected, given they are imperfect hosts. However, the majority of the sampled cats were under two years old (62.62%). Since our results demonstrated that age is a strong predictor of canine dirofilariosis, many of these animals may have been too young to have been exposed to infection. Nevertheless, it is crucial to continue screening for the infection in cats, as it has been previously described in the region [10,42,43] and its suburbs [44,45]. The diagnosis of dirofilariosis is especially challenging in cats, which are often amicrofilaraemic, making parasitological detection ineffective [46]. As a result, diagnosis relies almost exclusively on an integrated approach based on echocardiography, thoracic radiography and two serological tests, one for species-specific antigen and the other for serum antibody [46]. Serum antibody tests have greater sensitivity than antigen tests and so, in the event of a positive result, are a more useful diagnostic tool for raising the index of suspicion of infection in this host species. However, discerning between current and previous infections requires further clinical and laboratory examinations [46]. Unfortunately, commercial serological tests are rarely available to Brazilian veterinary practitioners [13], making definitive diagnosis difficult to achieve. When present, clinical signs of HARD are frequently mistaken for feline asthma [47], leading to misdiagnosis and often a fatal outcome. Thus, the use of this integrated approach for the detection of *D. immitis* infection is highly beneficial and could be implemented in the country. Monitoring the prevalence of this parasite among the feline population may also help prevent mortality by raising awareness among veterinarians and pet owners.

While *D. repens* was not detected in this study, this was expected considering the limited number of reports of the parasite in South America [15,18,48], which together suggest that it is not endemic in this area. Although *D. repens* is generally considered to be less pathogenic than *D. immitis*, it is important from a public health perspective, particularly due to its involvement in human ocular infection [49]. Therefore, active surveillance for this pathogen remains essential, especially in areas such as Rio de Janeiro, where continuous tourism and an ongoing influx of pets from endemic areas may facilitate its introduction. As previously reported [50], age was a significant predictor of *D. immitis* infection. Due to the long prepatent period, dogs younger than two years old are likely to be negative in diagnostic tests, with the odds increasing in older dogs (>4 years old), and this may be attributed to cumulative exposure to the parasite. A previous study strongly indicated that medium-sized dogs with short, dark fur were more likely to be infected [51]. However, in our study, there was no evidence that the size of the animal (*p* = 0.237) or its coat characteristics, such as coat length (*p* = 0.317) or colour (*p* = 0.342), significantly influences the probability of *D. immitis* infection.

Macrocyclic lactone usage was higher in urban areas, suggesting that preventive measures may be more effectively applied in the larger urban centre compared to the suburban regions. This difference may be due to the lack of disease awareness among the citizens of some suburban communities which can be explained by the scarcity of veterinary services provided in those areas. According to the official website of the Rio de Janeiro City Hall, there are 179 licensed veterinary clinics and two public hospitals that serve the companion animals of the city’s six million residents across an area of 1200.3 km^2^. In contrast, the suburban area, covering 43,969 km^2^, has only one public veterinary hospital and no official information is available regarding private veterinary clinics. Nonetheless, despite official data being unavailable, veterinary practices are known to be present in the territory [31]. Cost constraints and limited access to veterinary care are likely to lead to a more limited use of prophylaxis, which may contribute to the higher prevalence observed in the suburban area. However, it is important to note that the owners provided this information, so no evidence was available to determine whether a proper prophylactic regimen had been performed in each case prior to the study. Therefore, the results may not fully reflect the impact of macrocyclic lactones on *D. immitis* infection, and the findings are insufficiently robust to draw definitive conclusions.

This study presented a few limitations. Firstly, the animals enrolled in this study might not have been representative of the populations living in the study area. Due to the enrollment strategy adopted, animals tested in this study might have been from a specific socioeconomic background as the neutering programme was only open to owners with low-income status. The authors could not assess the effect of potential enrollment bias on study outcomes due to limited time available to gather socioeconomic information from each animal owner. Secondly, the information provided by owners through the questionnaire has limited reliability. Recall and social-desirability bias might have affected the accuracy of some of the responses (e.g., ML administration and pet lifestyle). Finally, serum antibody tests for feline samples were not available at our facilities at the time of testing, so no evidence of past/recent exposure or single sex infections with *D. immitis* could be gathered for this species.

Understanding the evolving dynamics of mosquito-parasite-host-environment interactions is key to enabling timely interventions, since environmental changes may impact mosquito survival and distribution. The rising prevalence of *D. immitis* in suburban areas highlights an emerging risk in regions previously considered to be non-endemic. Environmental factors, socioeconomic vulnerabilities, and limited access to veterinary care may together contribute to the spread of the disease. It is possible that these findings may be extrapolated to other suburban areas previously considered non-endemic, although further research is needed to confirm this hypothesis. Additionally, challenges in diagnosing infections in cats, and the potential presence of other filarial species call for effective ongoing surveillance. A more holistic approach, integrating environmental management, socioeconomic considerations, and veterinary care may be required to better understand and address the emergence of *D. immitis* in these areas.

## 5. Conclusions

This study demonstrates that combining test methods for detecting *Dirofilaria* spp. infection is critical for accurate disease diagnosis. The effectiveness of this integrated approach underscores the need for its implementation in veterinary practice, thereby generating reliable passive surveillance data.

The prevalence of *D. immitis* infection in dogs is increasing in areas previously considered non-endemic. This is driven by environmental and socioeconomic vulnerabilities in suburban regions, which favour vector survival and perpetuation. Furthermore, age continues to be the most reliable predictor for *D. immitis* infection. Sustained research, regular monitoring, and targeted interventions are essential to limit the spread of this zoonotic disease in neglected locations and protect both animals and humans.

## Figures and Tables

**Table 1:** Geographic distribution and prevalence of *D. immitis* infection among the 497 dogs and 107 cats.

Region	Municipality	Dogs			Cats	
		n	Positives	(%)	n	Positives
Suburban area	Belford Roxo	7	0	0.00	14	0
	Duque de Caxias	32	8	25	6	0
	Itaguaí	1	0	0.00	NA	NA
	Magé	1	0	0.00	NA	NA
	Mesquita	2	0	0.00	NA	NA
	Nova Iguaçú	8	0	0.00	1	0
	Seropédica	25	3	12	NA	NA
	Total	76	11	14.47	21	0
Urban area	Rio de Janeiro	421	26	6.17	86	0

% = Prevalence; NA = Not available.

**Table 2:** Univariable and multivariable analysis of risk factors associated with *D. immitis* infection in dogs including region, breed, sex, age, size, coat colour and length, access to outdoor spaces, time in current residence, and administration of preventive treatment.

Risk Factors	Negatives		Positives		Total	Prevalence (%)	Univariable		Multivariable	
	n	%	n	%			OR (CI 95%)	*p* Value	Adjusted OR (CI 95%)	*p* Value
**Region**										
Suburban area	65	14.13	11	29.73	76	14.47	Ref	0.020	Ref	0.003
Urban area	395	85.87	26	70.27	421	6.17	0.39 (0.19–0.86)		0.28 (0.13–0.64)	
**Breed**										
Mixed	243	52.83	17	45.95	260	6.54	Ref	0.420	NC	
Specific	217	47.17	20	54.05	237	8.44	1.32 (0.67–2.61)			
**Sex**										
Female	294	63.91	21	56.76	315	6.67	Ref	0.390	NC	
Male	166	36.09	16	43.24	182	6.75	1.35 (0.68–2.65)			
**Age**										
<2 yo	226	49.13	7	18.92	233	3.00	Ref	<0.001	Ref	<0.001
2–4 yo	127	27.61	11	29.73	138	7.97	2.80 (1.07–7.77)		2.83 (1.08–7.94)	
>4 yo	107	23.26	19	51.35	126	15.08	5.73 (2.44–15.06)		7.12 (2.94–19.33)	
**Size**										
Small	226	49.13	13	35.14	239	5.44	Ref	0.237	NC	
Medium	198	43.04	21	56.76	219	9.59	1.84 (0.91–3.87)			
Large	36	7.83	3	8.11	39	7.69	1.45 (0.32–4.77)			
**Coat Colour**										
Dark	211	45.87	14	37.84	225	6.22	Ref	0.342	NC	
Light	249	54.13	23	62.16	272	8.46	1.39 (0.71–2.84)			
**Coat Length**										
Short	247	53.70	23	62.16	270	8.52	Ref	0.317	NC	
Medium-Long	213	46.30	14	37.84	227	6.17	0.71 (0.35–1.39)			
**Access to outdoor spaces**										
Indoor	146	31.74	11	29.73	157	7.01	Ref	0.799	NC	
Outdoor	314	68.26	26	70.27	340	7.65	1.10 (0.54–2.37)			
**Time in current residence**										
Short	283	61.52	16	43.24	299	5.35	Ref	0.031	NC	
Long	177	38.48	21	56.76	198	10.61	2.10 (1.07–4.19)			
Administration of preventive treatment *										
No	270	62.36	25	73.53	295	8.48	Ref	0.183	NC	
Yes	163	37.64	9	26.47	172	5.23	0.60 (0.26–1.27)			

OR = Odds ratio; CI = Confidence interval; Ref = Reference; yo = Years old; NC: Not calculated; * Information not provided for 30 dogs.

## Data Availability

All the data presented in this study are included in the manuscript and Supplementary Materials. Further inquiries can be directed to the corresponding author.

## Supplementary Materials

The following supporting information can be downloaded at: https://www.mdpi.com/article/10.3390/vetsci12010003/s1, Supplementary Table S1. Parasitological, serological and molecular test results of the 37 *Dirofilaria immitis* positive canine samples including microfilariae measurements (mean ± SD), ELISA absorbances, Ct values, melting curve temperatures and Sanger sequencing results.
