# Supplementary material for: Microgeographical Variation in Dirofilaria immitis Prevalence in Dogs in Suburban and Urban Areas of Rio De Janeiro, Brazil
Source: Vet Sci. 2024 Dec 27;12(1):3. doi: 10.3390/vetsci12010003 (PMC11769065; doi:10.3390/vetsci12010003)
Supplement: Supplementary file 1 [file vetsci-12-00003-s001.zip › vetsci-3364021-supplementary.pdf]

Supplementary Table S1. Parasitological, serological and molecular test results of the 37 *Dirofilaria immitis* positive canine samples including microfilariae measurements (mean ±SD), ELISA absorbances, Ct values, melting curve temperatures and Sanger sequencing results.

Sample number	Knott's test				ELISA		Molecular analysis							
							Duplex qPCR			*A. reconditum* (cPCR)	12S cPCR			
	Result	Mf/ml	Length (µm)	Width (µm)	Result	Abs (nm)	Result	Ct	Tm (°C)	Result	Result	Sequencing	BLAST % identity	Accession number
1	Pos	3	NM	NM	Pos	0.77	Pos	20.9	76.0	Neg	NA	NA	NA	NA
2	Neg	0	NA	NA	Pos	0.25	Neg	NA	NA	Neg	NA	NA	NA	NA
3	Neg	0	NA	NA	Pos	0.32	Neg	NA	NA	Neg	NA	NA	NA	NA
4	NA	NA	NA	NA	Neg	0.05	Pos	31.9	76.0	Neg	Pos*	Not found	NA	NA
5	Pos	836	278.20 ± 5.2	6.12 ± 0.13	Pos	1.59	Pos	19.5	76.0	Neg	NA	NA	NA	NA
6	Pos	494	298.63 ± 4.9	6.01 ± 0.11	Pos	2.44	Pos	16.4	76.0	Neg	NA	NA	NA	NA
7	Pos	260	283.12 ± 5.0	5.99 ± 0.10	Pos	1.70	Pos	15.9	75.8	Neg	NA	NA	NA	NA
8	Pos	15	313.41 ± 5.4	6.13 ± 0.12	Pos	1.77	Pos	17.3	76.3	Neg	NA	NA	NA	NA
9	Pos	37	281.2 ± 4.8	6.03 ± 0.12	Neg	0.06	Pos	17.3	76.3	Neg	NA	NA	NA	NA
10	NA	NA	NA	NA	Pos	1.77	NA	NA	NA	NA	NA	NA	NA	NA
11	Pos	140	292.24 ± 5.1	6.01 ± 0.11	Pos	3.21	Pos	14.9	76.4	Neg	NA	NA	NA	NA
12	Neg	0	NA	NA	Neg	0.05	Pos	18.7	76.3	Neg	NA	NA	NA	NA
13	Neg	0	NA	NA	Pos	2.82	Pos	17.7	75.8	Neg	NA	NA	NA	NA
14	Pos	2	277.26 ± 5.3	5.53 ± 0.15	Neg	0.05	Neg	NA	NA	Neg	Pos	*D. immitis*	99.63%	KP898738.1
15	Pos	10	254.74 ± 4.7	4.67 ± 0.14	Pos	0.28	Neg	NA	NA	Pos	Pos	*D. immitis*	100%	OQ875916.1
16	Neg	0	NA	NA	Pos	1.90	Neg	NA	NA	Neg	NA	NA	NA	
17	NA	NA	NA	NA	Pos	0.21	Neg	NA	NA	Neg	NA	NA	NA	
18	NA	NA	NA	NA	Neg	0.05	Pos	31.5	76.3	Neg	Pos	*D. immitis*	99.48%	PP702284.1
19	Neg	0	NA	NA	Neg	0.06	Pos	20.2	75.8	Neg	NA	NA	NA	
20	Neg	0	NA	NA	Pos	1.79	Neg	NA	NA	Neg	NA	NA	NA	
21	NA	NA	NA	NA	Neg	0.05	Pos	22.9	76.8	Neg	NA	NA	NA	
22	Neg	0	NA	NA	Pos	1.51	Pos	19.5	75.3	Neg	NA	NA	NA	
23	Pos	300	311.22 ± 5.5	6.38 ± 0.16	Pos	0.84	Pos	14.5	75.5	Neg	NA	NA	NA	
24	NA	NA	NA	NA	Pos	1.53	Pos	23.0	76.5	Neg	NA	NA	NA	
25	Neg	0	NA	NA	Pos	0.85	Neg	NA	NA	Neg	NA	NA	NA	
26	Pos	>4000	281.49 ± 4.9	6.18 ± 0.13	Pos	0.34	Pos	18.8	75.5	Neg	NA	NA	NA	
27	Pos	>2500	296.78 ± 5.0	5.81 ± 0.14	Pos	0.28	Pos	18.8	76	Neg	NA	NA	NA	
28	Neg	0	NA	NA	Pos	1.35	Neg	NA	NA	Neg	NA	NA	NA	
29	Neg	0	NA	NA	Pos	0.38	Neg	NA	NA	Neg	NA	NA	NA	
30	NA	NA	NA	NA	Pos	0.26	Neg	NA	NA	Neg	NA	NA	NA	
31	Neg	0	NA	NA	Pos	0.85	Pos	23.8	76.8	Neg	NA	NA	NA	

32	Neg	0	NA	NA	Pos	0.41	Neg	NA	NA	Neg	NA	NA	NA
33	Neg	0	NA	NA	Pos	0.60	Neg	NA	NA	Neg	NA	NA	NA
34	Neg	0	NA	NA	Pos	0.20	Neg	NA	NA	Neg	NA	NA	NA
35	NA	NA	NA	NA	Neg	0.06	Pos	15.8	76.4	Neg	NA	NA	NA
36	Neg	0	NA	NA	Pos	0.45	Pos	28.4	76.9	Neg	NA	NA	NA
37	Neg	0	NA	NA	Pos	1.74	Pos	33.6	76.8	Neg	NA	NA	NA

Mf=Microfilariae; Abs=Absorbance; Pos=Positive; Neg=Negative; NM=Not measured; NA=Not applicable; **Cox1* cPCR; BLAST=Basic Local Alignment Search Tool
